# Preoperative Albumin-Bilirubin Score as a Predictor of Postoperative Complications After Liver Resection

**DOI:** 10.7759/cureus.111661

**Published:** 2026-06-28

**Authors:** Anupam Debnath, Bidhan C Das, Murshidul Arefin, Md Kawsar Bhuiyan, Golam Mahmud Rayhan, Nur Alam Mohim, Amit Chowdhury, Azfar Bin Anis, Ondrila Debnath Oishi, Mohsen Chowdhury

**Affiliations:** 1 Hepatobiliary Surgery, Evercare Hospital, Dhaka, BGD; 2 Hepatobiliary, Pancreatic and Liver Transplant Surgery, Bangladesh Medical University, Dhaka, BGD; 3 Surgery, National Gastroliver Institute and Hospital, Dhaka, BGD; 4 Surgery, Rupganj Upzilla Health Complex, Dhaka, BGD; 5 Surgery, Bangladesh Medical University, Dhaka, BGD; 6 Surgery, Mymensingh Medical College Hospital, Dhaka, BGD; 7 Surgery, United Medical College, Dhaka, BGD

**Keywords:** albi, ctp, liver resection, meld, posthepatectomy liver failure

## Abstract

Introduction: Hepatic resection is an important curative procedure for both benign and malignant liver diseases, and the risks associated with liver resection, especially posthepatectomy liver failure (PHLF), are still a major concern. This is the reason why it is clear that the correct evaluation of hepatic functional reserve is imperative before surgery. The conventional scoring systems, like Model for End-stage Liver Disease (MELD) and Child-Turcotte-Pugh (CTP), are less effective in predicting postoperative morbidity. An objective, inexpensive method, the albumin-bilirubin (ALBI) score, involving serum albumin and bilirubin, can be helpful in a resource-limited setting.

Objective: The objective of this study was to determine the preoperative ALBI score for predicting postoperative complications of patients undergoing hepatic resection.

Materials and methods: This is a prospective observational study carried out in the Department of Hepatobiliary, Pancreatic and Liver Transplant Surgery (HBLT) of Bangabandhu Sheikh Mujib Medical University (now Bangladesh Medical University (BMU)), Dhaka, from August 2023 till July 2024. The number of adult patients who underwent liver resection and met the inclusion criteria was 70, who were sampled purposively. All of the demographic, laboratory, clinical, and operative data were documented preoperatively. Presurgery scores for ALBI, CTP, and MELD were calculated. The postoperative complications were assessed within 30 days. Data were analyzed with Statistical Package for the Social Sciences (SPSS) version 23, and receiver operating characteristic curve (ROC) analysis was used to assess the predictive accuracy of ALBI.

Results: Out of 70 patients, 30 patients (42.9%) had postoperative complications. Patients with postoperative complications were significantly older than those without complications (50.67 ± 7.06 vs. 44.68 ± 10.26 years; *P* = 0.005). Biochemical parameters such as serum albumin were significantly decreased in the patients with complications. Liver failure after liver resection was significantly more common with an increase in the ALBI grade and did not occur in ALBI-1 (0.0%); there were 38.5% in ALBI-2 and 100% in ALBI-3. The ALBI score was significantly different between complication and non-complication groups, while the MELD score did not differ significantly between groups, whereas the CTP class showed a statistically significant difference. ALBI score showed weak-to-moderate predictive ability with area under the curve (AUC) 0.659, sensitivity 70.0%, and specificity 62.5% at a cut-off of -2.67 and in binary logistic regression analysis, age and ALBI score were independently associated with postoperative complications.

Conclusion: Preoperative ALBI score was significantly associated with postoperative complications and showed weak-to-moderate discriminatory ability. ALBI as having weak-to-moderate discriminatory ability and as a possible adjunctive marker, not a strong predictive test or replacement for clinical judgment and established scoring systems, but larger multicenter studies are needed to validate its cut-off and clinical usefulness.

## Introduction

Hepatic resection has long been considered the most important curative intervention to be applied to patients with benign and malignant liver pathology, and its clinical outcome has significantly improved with the development of surgical technology and perioperative care [1,2]. Even with these improvements, hepatectomy remains associated with a significant risk of postoperative morbidity and mortality, especially in people who have a pre-existing chronic liver disease such as cirrhosis and advanced hepatic fibrosis [3,4]. The most feared complication post hepatectomy is posthepatectomy liver failure (PHLF), which is a potentially fatal condition that is a result of the inadequate residual liver function following the resection [2]. The most important thing is, therefore, the accurate preoperative stratification of hepatic functional reserve to guide surgical decision-making, patient selection, and the minimization of risks.

Several instruments have been utilized to measure liver function. Traditional scores, such as the Child-Pugh score, the Model for End-Stage Liver Disease (MELD), and the Aspartate Aminotransferase to Platelet Ratio Index (APRI) score, are still widely used [5]. Other modalities like indocyanine green (ICG) retention testing, CT liver volumetry, technetium-99m scintigraphy, and preoperative portal pressure measurement have reported prognostic value [6,7]. The use of transient elastography liver stiffness measurement (LSM) is validated but expensive [8]. Both MELD and CTP have their limitations: The subgroup meta-analysis of eight studies demonstrated that the Child-Pugh score had a higher mean area under the receiver operating characteristic curve (AUROC) than the MELD score in predicting 12-month mortality; Child-Pugh is not the best method to predict morbidity [9]; and liver biopsy is an accurate method but is invasive [10].

Against this background, the albumin-bilirubin (ALBI) score has emerged as a promising objective marker. Developed in Japan in 2015, it is calculated from serum albumin and bilirubin alone, enabling objective determination from a single routine blood test [11]. It has been confirmed in primary biliary cirrhosis, hepatocellular carcinoma (HCC), and post-transplantation survival [11,12]. More recently, it is an effective predictor of PHLF and overall complications, and it has a better discriminative ability in certain settings [13]. The difference in the reported optimal cut-off values could be due to geographical and etiological variations [14]. The ALBI score is specifically well-suited in resource-constrained settings since albumin and bilirubin assays are both inexpensive and non-invasive [15]. There are some studies about the use of ALBI in some liver diseases but not much information on preoperative hepatic risk stratification in Bangladesh [16,17].

The current study thus aimed to assess the ability of the preoperative ALBI score to predict 30-day postoperative complications, particularly liver-related complications, following hepatic resection in a tertiary care hospital in Bangladesh.

## Materials and methods

Study design and setting

This prospective observational study was carried out from August 2023 to July 2024 for one year in the Department of Hepatobiliary, Pancreatic and Liver Transplant Surgery (HBLT) at Bangabandhu Sheikh Mujib Medical University (now Bangladesh Medical University (BMU)) at Shahbag, Dhaka, Bangladesh.

Study population

All patients who underwent liver resection during the study period in the HBLT, Bangabandhu Sheikh Mujib Medical University, Dhaka, who met the inclusion and exclusion criteria, were included in the study population.

Sample size determination

Sample size was determined according to the "Buderer formula" for diagnostic test studies. Sensitivity of the ALBI score was estimated at 90% based on a previous study carried out by Zhang et al. [18] and a reference proportion of postoperative outcomes of 50%. The final sample size was 70 when taking into account 10% dropouts or non-response with a 95% confidence level and precision of 10%. The purposive sampling technique was used.

Sampling method

The sampling technique was purposive, where participants were chosen following inclusion and exclusion criteria. This was a pragmatic single-center sample of eligible surgical patients, and that the findings should be generalized cautiously because referral patterns, case mix, and perioperative practice may differ across institutions.

Enrollment criteria

Patients included were adults who were undergoing liver resection. Patients with obstructive jaundice who had not been decompressed prior to surgery, had hemolytic jaundice, had renal dysfunction, had protein-losing enteropathy, and had severe malnutrition were excluded. Ethical clearance was obtained from the Institutional Review Board (IRB) of Bangabandhu Sheikh Mujib Medical University, Dhaka, Bangladesh (Ref. no. BSMMU/2023/12329), and written informed consent was obtained from all subjects before data collection.

**Figure 1:**
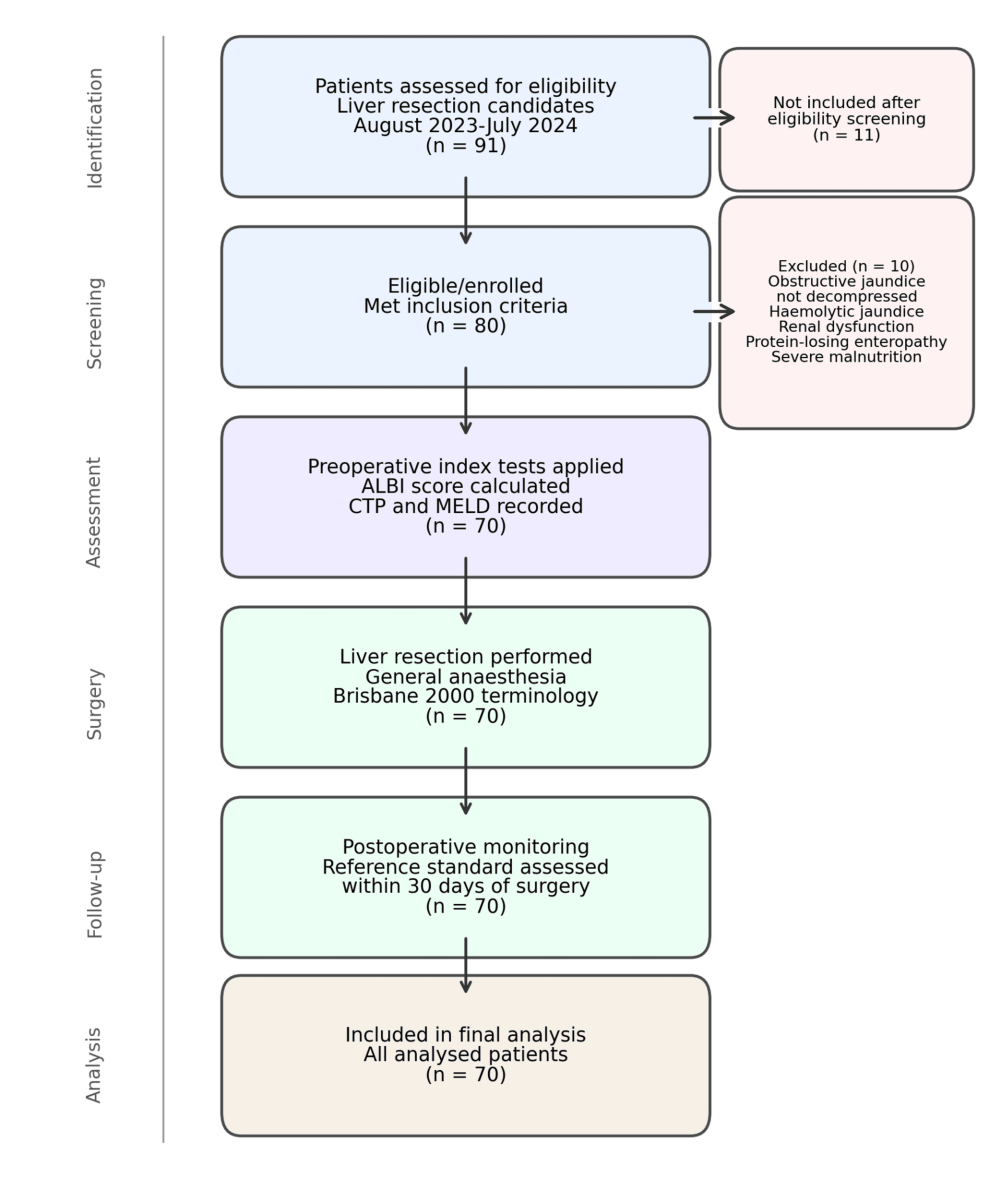
CONSORT-style study flow diagram of patients evaluated for the association between preoperative ALBI score and postoperative complications Created using Python 3.11 Matplotlib CONSORT: Consolidated Standards of Reporting Trials; ALBI: Albumin-bilirubin

The study enrollment and analysis pathway is presented in Figure 1.

Study procedure

All enrolled patients underwent detailed evaluation through history-taking, physical examination, laboratory investigations, and preoperative imaging. The ALBI score was calculated at admission using the published ALBI formula [19], and Child-Turcotte-Pugh (CTP) and MELD scores were recorded according to published scoring systems [20,21]. The ALBI score was calculated using the formula

$(\log10\ bilirubin\ &times;\ 0.66)+(albumin\ &times;\ &minus;0.085)$

Bilirubin was expressed in µmol/L and albumin in g/L for ALBI calculation. Bilirubin values measured in mg/dL were converted to µmol/L by multiplying by 17.1. ALBI groups were categorized as ALBI 1 ≤ -2.60; ALBI 2 > -2.60 to ≤ -1.39; ALBI 3 > -1.39 [19]. Patients were optimized before surgery to improve performance status and ensure biochemical parameters were suitable for anesthesia.

All liver resections were performed under general anesthesia using a standardized approach. Brisbane 2000 terminology for liver anatomy and resection was followed [22]. Abdominal access was achieved through a bilateral subcostal, modified Makuuchi, or inverted T incision, depending on surgeon preference. The abdominal cavity and liver were examined carefully, and liver parenchymal transection was performed with attention to hemostasis and prevention of bile leakage. Resected specimens were sent for histopathological examination. Postoperatively, patients were monitored in the ward, and biochemical markers were assessed early after surgery and repeated when clinically indicated.

Study variables

Demographics were age (years) and sex. Laboratory parameters before operation were hemoglobin, white blood cell count (WBC), platelet count, serum bilirubin, serum glutamic pyruvic transaminase (SGPT), serum glutamic-oxaloacetic transaminase (SGOT), serum albumin, international normalized ratio (INR) and serum creatinine. CTP score, MELD score, ALBI score, and ALBI grade (ALBI 1, ALBI 2, and ALBI 3) were variables used in liver function assessment before the operation. Intraoperative variables were the operation time (OT) in minutes, the amount of blood loss in milliliters, the transfusion of whole blood and the extent of liver resection (major liver resection or minor liver resection). The primary outcome variable was the status of postoperative complications or their absence, defined as "complications present" (PHLF, biliary fistula, pneumonia, atelectasis, wound infection, ascites, intra-abdominal abscess, and wound dehiscence) and the absence of complications. PHLF was defined as elevated INR with immune reconstitution inflammatory syndrome (IRIS) (hyperbilirubinemia) on or after postoperative day 5. The criteria for diagnosis of biliary fistula were: (1) bilirubin level > 3 times the serum bilirubin level after postoperative day 3; or (2) the need for intervention for either the collection of bilirubin or peritonitis. Posthepatectomy hemorrhage is defined as a drop in hemoglobin level by more than 3 g/dL from the postoperative baseline, transfusion due to decreasing hemoglobin or intervention to stop bleeding. Pneumonia was defined as the presence of clinical features with radiological evidence of pulmonary infection. Surgical site infection that caused raised temperature, discharge, erythema, and tenderness, which was treated with antibiotics, drainage, and wound intervention, was defined as a wound infection. Intraperitoneal fluids after surgery that needed to be treated were evaluated as ascites. Intra-abdominal abscess was defined as the presence of a radiological or operative collection which required antibiotics/drainage/intervention. Wound dehiscence was defined as a partial or complete separation of the surgical wound, which might need conservative or surgical intervention.

Data collection

We used a structured checklist to collect data, which covered all the variables of interest. Data were collected based on the record files of patients, history taking, clinical examination, observations during the perioperative and postoperative periods, and an assessment of biochemical markers in patients. The presentation of data and results was in the form of text with tables, figures, graphs, and diagrams where necessary. The utmost confidentiality and ethical principles were upheld during data storage and analysis.

Statistical analysis

Data were compiled in Microsoft Excel 2019 and analyzed using Statistical Package for the Social Sciences (SPSS) version 23. Categorical variables were presented as frequencies and percentages, while continuous variables were expressed as mean ± SD or median with interquartile range (IQR). Chi-square, Fisher’s exact test, Student’s t-test, and Mann-Whitney U-test were applied as appropriate. A p-value below 0.05 was considered statistically significant. Confidentiality and ethical principles were maintained throughout the study.

Ethical considerations

The ethical approval of this study was provided by the IRB of Bangabandhu Sheikh Mujib Medical University, Dhaka, Bangladesh (Ref. no. BSMMU/2023/12329). All participants had informed written consent before data collection, and strict confidentiality was maintained throughout the study.

## Results

The total number of patients who participated was 70, and 30 (42.9%) experienced postoperative complications. Patients with postoperative complications were significantly older than those without complications (50.67 ± 7.06 vs. 44.68 ± 10.26 years; *P* = 0.005). The laboratory parameters measured were not significantly different between the two groups except for serum albumin, which was significantly lower in the complication group (38 vs. 42 g/L; *P* = 0.016) (Table 1).

**Table 1:** Baseline characteristics of study participants (N=70) Values are presented as median (Q1-Q3) or mean ± SD, as appropriate. Test statistic column shows t for Student’s t-test, χ² for chi-square test, and U for Mann-Whitney U-test; p < 0.05 was considered statistically significant. WBC: White blood cell count; IQR: Interquartile range; SGPT: Serum glutamic pyruvic transaminase; SGOT: Serum glutamic-oxaloacetic transaminase; INR: International normalized ratio

Variables	Complication Present (n=30)	Complication Absent (n=40)	p-value	Test Statistic
Age (Years), Mean± SD	50.67±7.06	44.68±10.26	0.005	t = 2.892
Gender				
Male, n(%)	14 (46.7)	24 (60.0)	0.268	χ² = 1.23
Female, n(%)	16 (53.3)	16 (40.0)		
Preoperative Hemoglobin (g/dL), mean ±SD	11.76 ± 1.35	11.60 ± 1.04	0.585	t = 0.528
Preoperative WBC (10^9^/L), median (IQR)	8,000 (7,000-9,300)	8,400 (7,000-9,300)	0.807	U = 579.5
Preoperative Platelet (10^9^/L), median (IQR)	291,500 (272,250-335,000)	299,000 (260,000-330,000)	0.757	U = 574.0
Preoperative Bilirubin (mg/dL), median (IQR)	0.78 (0.43–1.08)	0.66 (0.48–0.91)	0.601	U = 556.0
Preoperative SGPT (U/L), median (IQR)	29 (25.5-75.0)	26.5 (24.0-42.0)	0.14	U = 476.0
Preoperative SGOT (U/L), median (IQR)	30 (22.2-59.5)	26.5 (21-33.8)	0.131	U = 473.0
Preoperative Albumin (g/L), median (IQR)	38 (33.2-41.5)	42 (39-43.2)	0.016	U = 398.0
Preoperative INR, median (IQR)	1.07 (1.0-1.27)	1.09 (1.0-1.2)	0.765	U = 575.0
Preoperative Creatinine, (mg/dL), median (IQR)	0.85 (0.64-1.07)	0.81 (0.62-1)	0.713	U = 569.0

**Figure 2:**
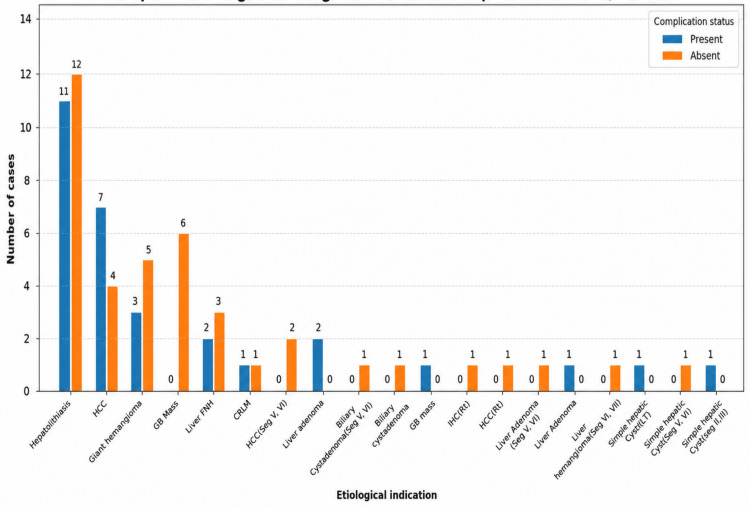
Etiological indication of surgery according to presence of complication (N=70) Created using Python 3.11 Matplotlib HCC: Hepatocellular carcinoma; GB: Gall bladder; CRLM: Colorectal liver metastases; IHC: Intrahepatic cholangiocarcinoma

Hepatolithiasis was the most frequent indication, with 11 cases with complications and 12 without complications; HCC followed with seven complicated and four uncomplicated cases (Figure 2).

The ALBI score differed significantly between groups (p < 0.001), the CTP class showed a significant difference (P = 0.030), and the MELD score was non-significant (P = 0.237) (Table 2).

**Table 2:** Comparison of preoperative different scoring systems between two groups (n=70) Tests used: Fisher's exact test and Student’s t-test; p < 0.05 was considered statistically significant. For the CTP comparison, the χ² statistic is displayed for editorial completeness, while the p-value is from Fisher’s exact test because of sparse cells. ALBI: Albumin-bilirubin; CTP: Child-Turcotte-Pugh; MELD: Model for End-stage Liver Disease

Score	Complication Present (n=30)	Complication Absent (n=40)	p value	Test Statistic
CTP Score				
Class A (5-6 Points), n (%)	26 (86.7)	40 (100.0)	0.030	χ² = 5.657
Class B (7-9 Points), n (%)	4 (13.3)	0 (0.0)		
MELD Score				
Mean ± SD	8.23 ± 3.42	7.38 ± 2.23	0.237	t = 1.20
Range (Min, Max)	6, 19	6, 14		
ALBI Score				
Mean ± SD	-2.37 ± 0.65	-2.86 ± 0.32	<0.001	t = 3.74
Range (Min, Max)	-3.92, -1.32	-3.31, -2.09		

There were no differences between groups in any intraoperative variable such as operative time, blood loss, blood transfusion, and extent of resections (all P > 0.05) (Table 3).

**Table 3:** Intraoperative variables (N=70). Tests used: Chi-square test, Fisher's exact test, and Student’s t-test; p < 0.05 was considered statistically significant. OT: Operation time

Intraoperative Variables	Complication Present (n=30)	Complication Absent (n=40)	p-value	Test Statistic
OT Time (Minutes), Mean± SD	173.67 ± 29.18	173.75 ± 24.04	0.99	t = -0.01
Amount of Blood Loss (ml), Mean ± SD	173.33 ± 66.61	165.00 ± 83.36	0.644	t = 0.46
Whole Blood Transfusion, n (%)				
0	0 (0.0)	2 (5.0)	0.224	χ² = 2.99
1	18 (60.0)	28 (70.0)		
2	12 (40.0)	10 (25.0)		
Type of Liver Resection				
Major Liver Resection, n (%)	9 (30.0)	10 (25.0)	0.642	χ² = 0.22
Minor Liver Resection, n (%)	21 (70.0)	30 (75.0)		

Across ALBI groups, there was no patient with PHLF in Grade 1, 38.5% in Grade 2, and 100% in Grade 3 (P < 0.001); the proportion of patients who developed wound infection and ascites differed significantly between grades (P < 0.001 both). Other complications were not significantly associated with ALBI grade (Table 4).

**Table 4:** Postoperative complications in different ALBI groups (N=70) ALBI score was recalculated from preoperative bilirubin and albumin. Groups: ALBI 1 ≤ -2.60; ALBI 2 > -2.60 to ≤ -1.39; ALBI 3 > -1.39. The χ² statistic is displayed in a separate column; p-values are from exact tests for 2 x 3 tables because of sparse cells. Multiple complications were recorded in some individual patients. PHLF: Posthepatectomy liver failure; ALBI: Albumin-bilirubin

Complication	Total N=70 (N%)	ALBI 1 (n=42) n (%)	ALBI 2 (n=26) n (%)	ALBI 3 (n=2) n (%)	p-value	Test Statistic
PHLF	12 (17.1)	0 (0.0)	10 (38.5)	2 (100.0)	<0.001	χ² = 26.676
Biliary Fistula	4 (5.7)	2 (4.8)	2 (7.7)	0 (0.0)	0.827	χ² = 0.381
Pneumonia	3 (4.3)	2 (4.8)	1 (3.8)	0 (0.0)	0.939	χ² = 0.125
Atelectesis	4 (5.7)	2 (4.8)	2 (7.7)	0 (0.0)	0.827	χ² = 0.381
Wound Infection	15 (21.4)	2 (4.8)	11 (42.3)	2 (100.0)	<0.001	χ² = 20.995
Ascites	5 (7.1)	1 (2.4)	2 (7.7)	2 (100.0)	<0.001	χ² = 27.448
Intra-abdominal Abscess	1 (1.4)	0 (0.0)	1 (3.8)	0 (0.0)	0.424	χ² = 1.717
Wound Dehiscence	2 (2.9)	0 (0.0)	2 (7.7)	0 (0.0)	0.175	χ² = 3.484

In binary logistic regression analysis, age and ALBI score were independent risk factors for postoperative complications after liver resection. A higher ALBI score was significantly associated with increased odds of complications (adjusted odds ratio (OR): 6.403; 95% confidence interval (CI): 1.887-21.722; P= 0.003), while age was significantly associated with increased odds of postoperative complications after adjustment (adjusted OR: 1.0751; 95% CI: 0.003-1.153; P = 0.042). Male sex and MELD score were not statistically significant predictors in the adjusted model (Table 5).

**Table 5:** Factors associated with complication following liver resection according to binary logistic regression analysis (N=70) Binary logistic regression model with complication present as the outcome. OR: Odds ratio; CI: Confidence interval; MELD: Model for End-stage Liver Disease; ALBI: Albumin-bilirubin

Factors	Adjusted OR (95% CI)	p value
Age	1.075 (1.003-1.153)	0.042
Male sex	1.223 (0.376-3.983)	0.738
MELD score	1.017 (0.800-1.293)	0.891
ALBI score	6.403 (1.887-21.722)	0.003

**Figure 3:**
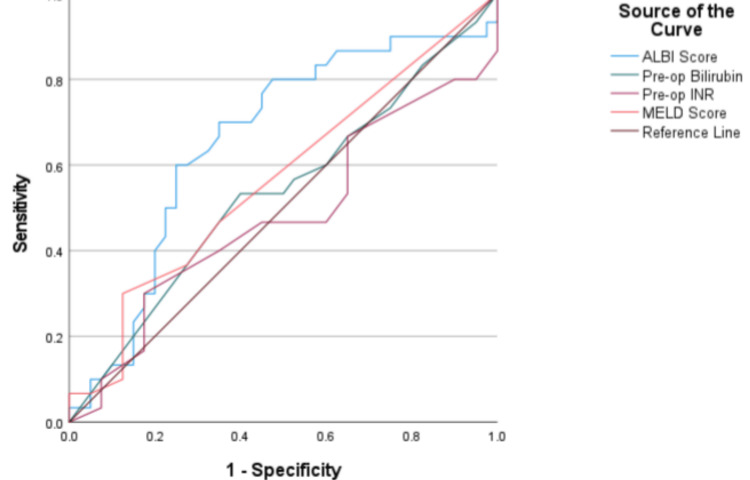
ROC curve of different biomarkers for predicting postoperative complication of liver resection (N=70) Created using Python 3.11 Matplotlib ROC: Receiver operating characteristic; ALBI: Albumin-bilirubin; INR: International normalized ratio; MELD: Model for End-stage Liver Disease; AUC: Area under the curve

Receiver operating characteristic curve (ROC) analysis showed that the ALBI score was a weak-to-moderate predictive factor for postoperative complications (area under the curve (AUC) 0.659; 95% CI: 0.505-0.792; P = 0.024) with the optimal cut-off, −2.67, having a sensitivity of 70.0% and specificity of 62.5% (Figure 3). Bilirubin, INR, and MELD score were not significant predictors (AUC 0.479-0.568; all P > 0.05) (Table 6).

**Table 6:** Cut-off values of preoperative markers used to predict postoperative complication of liver resection: sensitivity, specificity, Youden index and AUC for different cut-off values AUC: Area under the curve; CI: Confidence interval; ALBI: Albumin-bilirubin; INR: International normalized ratio; MELD: Model for End-stage Liver Disease

Marker	Cut-off	Sensitivity (%)	Specificity (%)	Youden Index	AUC	p value	95% CI
ALBI score	-2.67	70	62.5	0.325	0.659	0.024	0.505–0.792
Preoperative Bilirubin (mg/dL)	0.73	53.3	60	0.133	0.537	0.602	0.397–0.676
Preoperative INR	1.25	30	82.5	0.125	0.479	0.767	0.337–0.622
MELD score	9.5	46.7	87.5	0.175	0.568	0.336	0.430–0.705

## Discussion

In our study of 70 patients who were undergoing liver resection, 30 (42.9%) had postoperative complications. The patients with postoperative complications were significantly older than those without postoperative complications (50.67 ± 7.06 vs. 44.68 ± 10.26 years, P = 0.005). This finding is consistent with that of Tzeng et al. [23], who showed that elderly patients (age ≥ 75 years) were associated with an increased risk of severe complications and mortality following hepatectomy. In contrast, Okinaga et al. [24] found that operative risk rises with advancing age up to the 70s and then levels off in very elderly patients, likely due to the careful selection of older candidates for surgery. Therefore, age can play a role in the risks of the postoperative period, but the chronological age should be taken into account in combination with the physiological reserve, the presence of co-morbidities, liver function and the extent of the surgery.

A significant difference in serum albumin levels was observed between patients with and without complications (P = 0.016) before surgery, indicating poorer hepatic reserve in the patients with complications. Wound infection was significantly associated with ALBI grade (P = 0.044), and there was a significant gradient by ALBI grades, with 0% of Grade 1 cases, 35.5% of Grade 2 cases, and 100% of Grade 3 cases developing PHLF (P < 0.001). Conventional scoring systems, such as CTP and MELD, demonstrated subtle significance (P = 0.030 and P = 0.237, respectively), which is consistent with earlier findings that current scoring systems might not be able to identify modest hepatic dysfunction in individuals with mild to moderate hepatic insufficiency [25].

All of the intraoperative parameters (operative time (OT), blood loss, transfusion, and extent of resection), as well as tumor characteristics, were found to be similar among groups (all P > 0.05), suggesting that the extent of resection is not the most important determinant of complication in this study population but rather that of the hepatic reserve before surgery. The ALBI score was the only one to be statistically significant with an AUC of 0.659 (95% CI: 0.505-0.792; P = 0.024) and an optimal cut-off of −2.67; the sensitivity was 70.0%, and the specificity was 62.5%. This is similar to the AUC reported by Park et al. of 0.676 with a cut-off of -2.62 [26]. But compared to other previous studies, the AUC of the ALBI score in our study was lower than 0.782 by Zhang et al., 0.723 by Wang et al., and 0.745 by Zou et al. [18,27,28]. The modestly lower AUC in the present study could reflect differences in sample size or case mix because variations in ALBI scores are known to be influenced by population differences [26]. However, neither bilirubin (AUC 0.537) nor the MELD score (AUC 0.568) was significant. This illustrates the usefulness of the composite ALBI score in comparison to individual markers, as well as the more advanced risk categorization and increased predictive accuracy when the two values are combined [1,25]. This allows shifting the clinical care to patient optimization before surgery, particularly through patient selection through ALBI. Additionally, the ALBI score has been validated in different patients, such as HCC patients who receive trans-arterial chemoembolization and systemic therapy, further reinforcing the ALBI score as a universal hepatic marker [27-30]. 

There were several limitations in this study. It was carried out in one tertiary care hospital and had a relatively small number of patient samples, so the findings may not be generalizable. Finally, using purposive sampling may also cause selection bias. The assessment of the postoperative complication was done only within 30 days, and no delayed complications, long-term liver function, survival or recurrence was evaluated. The study used three different methods to assess liver function - ALBI, CTP, and MELD scores - but other ways of assessing liver function (such as ICG clearance, LSM, CT volumetry, or histological fibrosis grading) were not used. Additionally, there was clinical heterogeneity caused by the inclusion of different liver pathologies and liver resection types.

## Conclusions

Preoperative ALBI score was significantly associated with postoperative complications after hepatic resection and had weak-to-moderate discriminatory power in ROC analysis. Due to the small number of study participants in this single-center study and the model instability, ALBI should not be used as a stand-alone risk-stratification tool. It is objective and inexpensive and relies on laboratory parameters routinely available, though, and ALBI indicates exploratory evidence of association and potential adjunctive value in resource-limited settings. Its use may help identify patients who require closer perioperative optimization and postoperative monitoring. More large-scale multi-center studies are needed to establish an optimum cut-off value in Bangladeshi patients and the incorporation of ALBI into the prehepatic resection clinical decision process.
